# Higher body mass index and lower lateral femoral condyle index are associated with graft failure following adolescent anterior cruciate ligament reconstruction: A registry‐based 2‐year survival analysis

**DOI:** 10.1002/jeo2.70978

**Published:** 2026-11-11

**Authors:** Alexander H. Seeto, Kylie Bradford, David Bade, Liam Johnson, Sheanna Maine, David J. Saxby, Christopher P. Carty, Ivan P. Astori

**Affiliations:** ^1^ School of Medicine and Dentistry Griffith University Gold Coast Australia; ^2^ Australian Centre for Precision Health and Technology (PRECISE) Griffith University Gold Coast Australia; ^3^ Department of Orthopaedics Children's Health Queensland Hospital and Health Service Brisbane Australia

**Keywords:** adolescent, anterior cruciate ligament reconstruction, body mass index, graft failure, lateral femoral condyle index

## Abstract

**Purpose:**

Graft failure following anterior cruciate ligament reconstruction represents a serious clinical outcome in a young, active and skeletally developing population. This study aimed to identify risk factors for 2‐year graft failure following anterior cruciate ligament reconstruction in adolescents.

**Methods:**

Patients younger than 18 years undergoing complete or partial transphyseal anterior cruciate ligament reconstruction were recruited through a prospective paediatric anterior cruciate ligament registry. The primary outcome was graft failure, defined as confirmed graft rupture or clinical failure requiring repeat surgery for instability. Kaplan–Meier survival analysis and univariable followed by multivariable Cox proportional hazards regression were performed, with censoring at 2 years. Candidate variables included body mass index, skeletal maturity, meniscal compromise, mechanical axis deviation, lateral femoral condyle index, tibial slope and lateral extra‐articular tenodesis.

**Results:**

A total of 290 patients with a minimum 2‐year follow‐up were included. Twenty graft failures occurred, comprising eighteen confirmed ruptures and two clinical failures. Estimated 2‐year graft survival was 93.1% (95% confidence interval [CI]: 90.2%–96.1%). In the primary multivariable model, higher body mass index (BMI) (hazard ratio [HR]: 1.13; 95% CI: 1.05–1.22, *p* = 0.001) and lower lateral femoral condyle index (LFCI) (HR per 0.01‐unit increase: 0.93; 95% CI: 0.88–0.99, *p* = 0.018) were associated with failure. In an exploratory sensitivity analysis additionally adjusting for preoperative skeletal maturity, the associations of higher BMI (HR: 1.11, 95% CI: 1.02–1.20; *p* = 0.011) and lower LFCI (HR per 0.01‐unit increase: 0.919, 95% CI: 0.865–0.977; *p* = 0.006) with graft failure were maintained.

**Conclusion:**

Higher BMI and lower lateral femoral condyle index were associated with 2‐year graft failure following adolescent anterior cruciate ligament reconstruction, with these associations maintained in exploratory sensitivity analyses. Given the limited number of failure events, these findings should be considered exploratory and require validation in larger cohorts before application to individual risk prediction.

**Level of Evidence:**

Level II, prognostic study.

## Graphical abstract

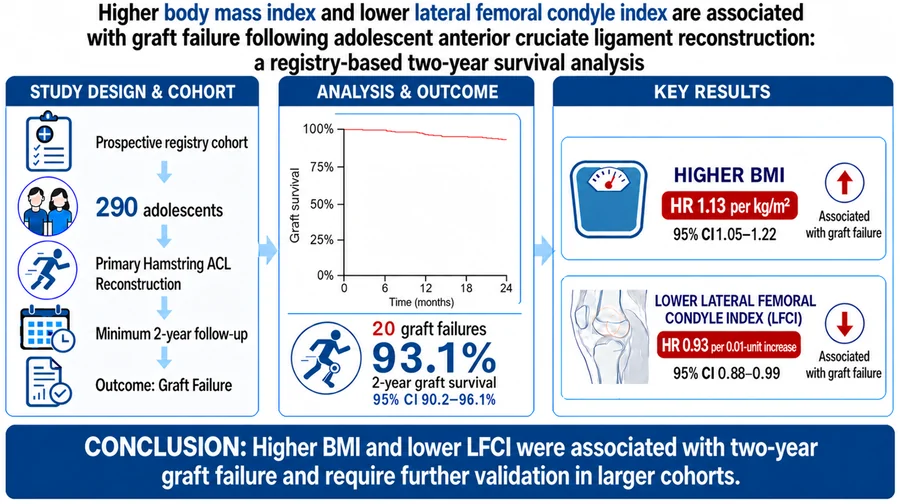

Higher body mass index and lower lateral femoral condyle index were associated with 2‐year graft failure and require validation in larger cohorts.

**Abbreviations**
ACL: anterior cruciate ligament
ACLR: anterior cruciate ligament reconstruction
AIC: Akaike information criterion
BMI: body mass index
CI: confidence interval
GNRB: Genourob arthrometer
HR: hazard ratio
HREC: Human Research Ethics Committee
LET: lateral extra‐articular tenodesis
LFCI: lateral femoral condyle index
LTS: lateral tibial slope
MAD: mechanical axis deviation
MRI: magnetic resonance imaging
MTS: medial tibial slope
PACS: picture archiving and communication system
QCH: Queensland Children's Hospital
REDCap: research electronic data capture
SD: standard deviation
WHO: World Health Organization

## INTRODUCTION

Graft failure represents a devastating outcome following anterior cruciate ligament (ACL) reconstruction (ACLR) in children, often resulting in prolonged rehabilitation, functional and psychological limitations and increased risk of early‐onset osteoarthritis [17, 20, 26]. Despite advances in surgical technique and rehabilitation protocols, the incidence of graft failure in the paediatric and adolescent population remains a significant clinical challenge, yet underlying risk factors remain incompletely defined [3, 13]. A systematic review and meta‐analysis of patients 19 years and younger undergoing ACLR reported pooled autograft failure rates of 10%–15% [4]. While anatomical factors have been implicated in the aetiology of primary ACL injuries in children and adolescents [5, 23], whether these same factors influence the risk of graft failure has not been systematically investigated.

Management of ACL injury in skeletally immature patients requires consideration of remaining growth, associated injuries and sporting demands [10]. Anatomical variations such as coronal plane valgus alignment, tibial slope and lateral femoral condyle index (LFCI) may alter joint biomechanics and influence rotational stability of the knee [2, 5, 14, 23, 24]. These morphological characteristics may similarly influence graft failure risk. Identifying anatomically high‐risk patients preoperatively may therefore assist risk stratification and inform adjunctive stabilisation, rehabilitation and return‐to‐sport strategies.

Registry‐based cohort studies provide a robust framework for evaluating graft outcomes and identifying risk factors in large, real‐world populations. The primary aim of this study was to describe the incidence of graft failure in a prospectively recruited registry cohort of adolescent patients following primary ACLR. The secondary aim was to explore the association between lower limb morphology, specifically mechanical axis deviation (MAD), LFCI, tibial slope and associated meniscal status and the risk of graft failure within 2 years of surgery. It was hypothesised that patient‐specific, knee morphological and surgical factors would be associated with graft failure within 2 years of adolescent ACL reconstruction.

## METHODS

### Participant recruitment

Institutional ethics approval for this study was received, and guardians of all participants provided written informed consent. The study participants were recruited prospectively from the single centre tertiary Queensland Children's Hospital (QCH) Paediatric ACL Injury Registry between September 2018 and January 2024. Patients were enrolled after outpatient orthopaedic consultations for their ACL injuries. Included patients were younger than 18 years of age and had sustained ACL injuries for which complete transphyseal or partial transphyseal ACLR was indicated. The age criterion reflected the pragmatic design of the paediatric ACL registry and its institutional eligibility criteria, rather than serving as a surrogate for skeletal age. Patients who underwent extra‐physeal ACLR were excluded. Other exclusion criteria included multi‐ligament knee injuries, previous ACLR, congenital limb abnormalities, bony or soft tissue pathology affecting anatomy (infection or trauma), bilateral ACL injuries and neurological disorders.

### Data collection and variable definitions

Demographic, clinical, operative and imaging data were prospectively recorded within the QCH Paediatric ACL Injury Registry and supplemented by review of the electronic medical record and operative documentation. Baseline variables included age at surgery, sex, body mass index (BMI), skeletal age, surgical technique, lateral extra‐articular tenodesis (LET), medial and lateral meniscal status and imaging‐derived morphological measurements.

Skeletal age was assessed on baseline preoperative knee magnetic resonance imaging (MRI) using the method described by Meza et al. [18]. Patients were stratified into two groups based on whether they had greater or less than 12 months of skeletal growth remaining. Greater than 12 months of growth remaining was defined as a skeletal age of <12 years in females or <15 years in males, corresponding to incomplete ossification of the tibial tubercle apophysis. It is important to note these measures were conducted preoperatively as a measure of estimated remaining growth.

Meniscal status was determined from intraoperative findings and recorded separately for the medial and lateral menisci. For the purposes of analysis, meniscal compromise was defined as a tear managed with debridement or left unrepaired because it was considered stable; intact menisci and repaired tears formed the comparison group. This composite was intended to capture possible tissue loss or persistent meniscal pathology, while recognising that debrided and untreated tears are clinically distinct. All four meniscal categories are reported separately in Table 1.

**Table 1:** Baseline variables.

Variable	Overall (*n* = 290)	No failure (*n* = 270)	Graft failure (*n* = 20)	*p*‐Value
Demographics				
Age, years (mean ± SD)	14.4 ± 1.8	14.4 ± 1.8	14.9 ± 1.1	0.229
Sex, female, *n* (%)	148 (51.0)	139 (51.5)	9 (45.0)	0.743
BMI, kg/m² (mean ± SD)	23.5 ± 5.1	23.2 ± 5.0	26.9 ± 5.7	**0.016**
Greater than 12 months skeletal growth remaining estimated preoperatively, *n* (%)	88 (30.3)	86 (31.9)	2 (10.0)	**0.044**
Surgical details				
Technique, *n* (%)				0.610
Transphyseal	277 (95.5)	257 (95.2)	20 (100.0)	
Partial transphyseal	13 (4.5)	13 (4.8)	0 (0.0)	
Graft Diameter after preparation, mm (mean ± SD)	8.1 ± 0.9	8.1 ± 0.9	8.4 ± 0.9	0.172
LET performed, *n* (%)	58 (20.0)	53 (19.6)	5 (25.0)	0.772
Graft type, *n* (%)				0.149
Semitendinosus	109 (37.6)	98 (36.3)	11 (55.0)	
Semitendinosus/Gracilis	181 (62.4)	172 (63.7)	9 (45.0)	
Intraoperative medial meniscal status, *n* (%)				**0.048**
No meniscal tear	211 (72.8)	200 (74.1)	11 (55.0)	
Repaired tear	48 (16.6)	44 (16.3)	4 (20.0)	
Debrided tear	17 (5.9)	13 (4.8)	4 (20.0)	
Untreated tear	14 (4.8)	13 (4.8)	1 (5.0)	
Medial meniscal compromise (debrided + untreated tears)	31 (10.7)	26 (9.6)	5 (25.0)	**0.049**
Intraoperative lateral meniscal status, *n* (%)				**0.012**
No meniscal tear	164 (56.6)	153 (56.7)	11 (55.0)	
Repaired tear	64 (22.1)	63 (23.3)	1 (5.0)	
Debrided tear	36 (12.4)	29 (10.7)	7 (35.0)	
Untreated tear	26 (9.0)	25 (9.3)	1 (5.0)	
Lateral meniscal compromise (debrided + untreated tears)	62 (21.4)	54 (20.0)	8 (40.0)	**0.047**
Baseline morphological parameters				
LFCI (mean ± SD)	0.70 ± 0.08	0.70 ± 0.08	0.66 ± 0.06	**0.046**
MAD, mm (median, IQR)	7.1 (0.0–12.4)	5.7 (0.6–11.5)	7.2 (−0.3 to 12.4)	0.766
LTS,° (mean ± SD)	7.1 ± 3.7	7.0 ± 3.6	8.7 ± 3.6	**0.044**
MTS,° (mean ± SD)	5.3 ± 3.3	5.2 ± 3.2	6.1 ± 4.2	0.251

*Note*: Patients were stratified into two groups of skeletal age, those with greater or less than 12 months of skeletal growth remaining defined by the methods of Meza et al. [18]. *P*‐values for meniscal‐status headings compare the four categories. *P*‐values for meniscal compromise compare debrided or untreated tears versus intact or repaired menisci. Normality was assessed using the Shapiro–Wilk method. Continuous data were recorded as mean and standard deviation and compared using *t*‐tests if parametric or as median and IQR and compared using Wilcoxon rank‐sum test if nonparametric. Categorical variables were recorded as frequency and percentage and were compared using Fisher's exact test. Bold *p*‐values indicate statistical significance (*p* < 0.05).

Abbreviations: BMI, body mass index; IQR, interquartile range; LET, lateral extra‐articular tenodesis; LFCI, lateral femoral condyle index; LTS, lateral tibial slope; MAD, mechanical axis deviation; MTS, medial tibial slope; SD, standard deviation.

### Measurement of morphological parameters

These measurement protocols have been previously described in detail and have demonstrated excellent intra and interrater reliability (Figure 1) [23, 24]. As previously detailed, all measurements were performed by a single clinician author (A.H.S.). To evaluate intrarater reliability, the primary measurer repeated measurements on a randomly generated subset of 15 patients 2 weeks after initial measurement. Interrater reliability was assessed on the same blinded randomly generated subset of 15 patients by the senior author (I.P.A.), a fellowship‐trained consultant paediatric orthopaedic surgeon. All reliability measurements were blinded to patient identity and group. Intra and interrater reliability (two‐way random with absolute agreement) was reported using intraclass correlation coefficients (ICC).

**Figure 1:**
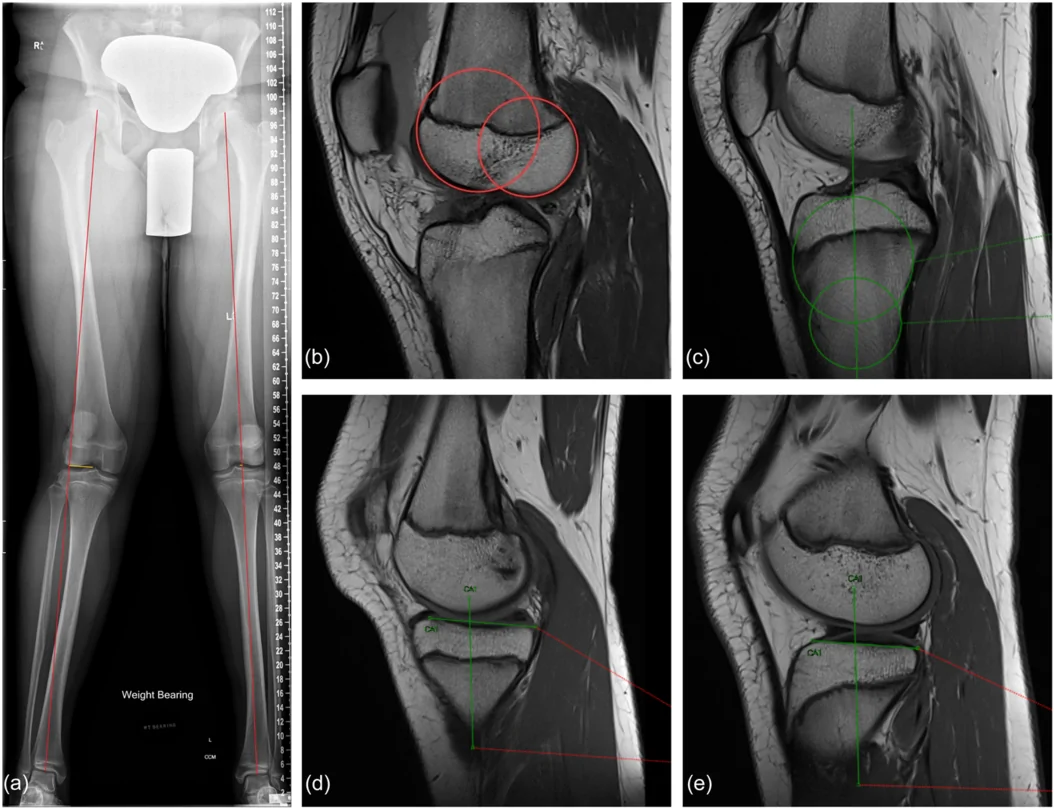
Measurement of lower‐limb alignment and knee morphological parameters. (a) Mechanical axis deviation (MAD) measured on a standing, weight‐bearing hip–knee–ankle radiograph as the perpendicular distance between the centre of the knee and the mechanical axis joining the centres of the femoral head and ankle. (b) Lateral femoral condyle index (LFCI) measured on sagittal magnetic resonance imaging as the ratio of the diameter of the posterior flexion circle to the diameter of the anterior extension circle. (c) Establishment of the longitudinal tibial reference axis using the two‐circle method described by Hudek et al. [15]. (d) Medial tibial slope (MTS) measured as the angle between a tangent to the medial tibial plateau and a line perpendicular to the longitudinal tibial reference axis. (e) Lateral tibial slope (LTS) measured using the corresponding tangent to the lateral tibial plateau.

Baseline weight‐bearing long‐leg alignment films were obtained preoperatively. Long‐leg radiographs were weight‐bearing, digitally stitched hip‐knee‐ankle teleoroentgenograms, standardised with forward‐facing patellae and knees in full extension. These radiographs were conducted when patients could maximally extend their knees and mobilise without aids. Mechanical axis deviation (MAD) was then calculated as the perpendicular distance between the knee centre and the mechanical axis, which was measured as the line joining the hip and ankle centres. Lateral MAD described a mechanical axis lateral to the knee centre, whereas medial MAD described a mechanical axis medial to the knee centre (Figure 1a).

Preoperative MRI of the injured limb was performed (1.5 T scanner, MAGNETOM Avantofit syngo MR VE 11‐B, Siemens) (3D PD SPACE sequence, slice thickness: 1.0 mm, voxel size: 0.83 × 0.83 × 1.0 mm). LFCI was measured using the method described by Hodel et al. [14]. Briefly, the midsagittal plane of the lateral condyle was identified at the level of the popliteal groove on the coronal plane, and the corresponding sagittal slice was identified. On the sagittal slice, the extension curvature of the femoral condyle was approximated by drawing an anterior circle fitted to the subchondral bone at the 6 and 9 o'clock positions. Similarly, the flexion curvature was approximated by a posterior circle fitted to the subchondral bone at the 6 and 3 o'clock positions. LFCI was calculated as the ratio of the diameter of the flexion circle to the diameter of the extension circle (Figure 1b), whereby a ratio closer to zero represents a smaller posterior/flexion diameter. A LFCI ratio of <0.70 has been previously identified as a cutoff for both ACL injury and graft failure in adults [14].

Medial tibial slope (MTS) and lateral tibial slope (LTS) were calculated according to the method outlined by Hudek et al. [15]. A longitudinal tibial reference axis was established using the two‐circle method (Figure 1c). MTS and LTS were then measured as the angle between the respective plateau tangent and a line perpendicular to this reference axis (Figure 1d–e).

### Surgical technique

All ACLR procedures were performed by one of four fellowship‐trained consultant paediatric orthopaedic surgeons (L.J., D.B., S.M., I.P.A.). In keeping with the pragmatic, real‐world design of the study, surgical technique, graft choice and fixation method were determined on a case‐by‐case basis according to individual clinical indications and surgeon preference. When appropriate, transphyseal approaches minimised physeal damage by avoiding the femoral perichondral ring and by orienting tibial and femoral tunnels more vertically and the tibial tunnel more centrally. All transphyseal procedures utilised hamstring soft‐tissue autografts. Partial transphyseal techniques were indicated in patients with 2–3 years of skeletal growth remaining and utilised hamstring soft‐tissue autografts with a transphyseal tibial tunnel and a physeal‐sparing femoral fixation via an anchor. Where possible, the menisci were preferentially repaired. LET was performed at the surgeon's discretion according to patient risk stratification, typically using a modified Lemaire or Ellison technique [6, 16] (Table 1).

### Outcome definition and ascertainment

The primary outcome was graft failure within 2 years of surgery, defined as either (1) confirmed graft rupture or (2) symptomatic clinical failure requiring repeat operative treatment for recurrent instability. Graft rupture was defined as disruption of the ACL graft confirmed on MRI or arthroscopy. Clinical failure was defined as persistent or recurrent symptomatic instability attributed to inadequate ACL reconstruction stability that resulted in repeat operative treatment, irrespective of graft continuity on imaging.

Objective residual laxity alone was not classified as graft failure in the absence of symptomatic instability requiring operative treatment. Accordingly, patients with a GNRB® arthrometer [22] side‐to‐side difference ≥5 mm or grade ≥2 pivot shift who remained clinically asymptomatic and did not require operative treatment were recorded separately but were not considered failure events.

The date of failure was defined as the date of the causative graft rupture when known. Where the precise date was unavailable, the date of the first clinical, imaging or operative assessment confirming failure was used. Given the inclusion of clinical failures within the composite primary outcome, a sensitivity analysis was performed restricting events to confirmed graft ruptures only.

### Follow‐up

Failure was ascertained through a multi‐modal approach to ensure comprehensive capture of events. Initial data collection was performed through the REDCap [12] electronic data capture tool within the QCH Paediatric ACL Injury Registry. This was supplemented with electronic medical record chart review linked to both the index hospital and other public hospitals across the state. Subsequently, the picture archiving and communication system (PACS) was interrogated for imaging data, including both local institutional PACS and community imaging facilities. As a final measure, patients were contacted directly via telephone to confirm follow‐up status and ascertain any additional failure events not captured through electronic systems. This multi‐pass approach ensured comprehensive identification of failure events occurring within the 2‐year follow‐up window. If no failure event occurred within 2 years of surgery, the patient's follow‐up was censored at 2 years.

### Statistical analysis

Time‐to‐event analysis was performed using Kaplan–Meier survival curves to estimate the probability of graft survival over the 2‐year follow‐up period. For participants who experienced graft failure, follow‐up time was calculated from the date of surgery to the date of failure. Participants without graft failure were censored at 2 years after surgery. Univariable Cox proportional hazards regression was conducted to assess each variable (age, sex, skeletal growth, BMI, LFCI, MAD, tibial slope parameters, meniscal compromise and LET) and the risk of failure. Continuous variables were modelled linearly to minimise degrees of freedom. The proportional hazards assumption was assessed for each covariate and for the overall Cox model using Schoenfeld residuals. Cox regression analyses used a common complete‐case cohort comprising participants with complete data for all candidate variables. The association between missing data and graft failure status was assessed using logistic regression for each variable with incomplete data, with event status, age and sex included as covariates. No multiplicity correction was applied because univariable analyses were exploratory and used to identify candidate variables. The sample size was determined by the eligible registry cohort. With 20 graft failure events, the primary multivariable model was restricted to two predictor parameters using a prespecified ten‐events‐per‐parameter criterion to limit model complexity. This criterion was not a formal power calculation. Candidate variables were prioritised according to strength of univariable association and biological plausibility, and the primary multivariable model was selected using the Akaike Information Criterion. Given the limit of two covariates in the primary model, the six possible two‐variable combinations of BMI, LFCI, LTS and medial meniscal compromise were compared using the Akaike information criterion. To assess the stability of hazard ratio estimates, profile likelihood confidence intervals (CIs) were calculated alongside standard Wald intervals for all Cox regression models. An exploratory sensitivity analysis repeated the primary model in the same complete‐case cohort, restricting the endpoint to confirmed graft rupture only. Clinical failures without confirmed rupture were censored at the date of clinical failure. Because skeletal growth was considered a biologically plausible potential confounder of the associations identified in the primary model, an additional exploratory sensitivity model was performed incorporating skeletal growth alongside the variables in the primary model. Given the limited number of failure events relative to the number of covariates, this analysis was considered exploratory and was used to assess the stability of the primary effect estimates rather than to establish additional independent variables. An additional analysis was conducted where BMI was substituted with age‐ and sex‐standardised BMI *z*‐scores to account for the developmental variation in body composition across the cohort's age range [21], calculated using World Health Organization (WHO) Child Growth Standards. Nevertheless, raw BMI was included as a primary continuous variable in preference to age‐ and sex‐standardised BMI *z*‐scores, as the mechanistically relevant quantity for graft loading was considered absolute body mass relative to height rather than population‐relative adiposity.

Time to graft failure was also explored across morphological subgroups of interest using dichotomised Kaplan‐Meier survival curves, defined by significant MRI thresholds for graft failure (LFCI < 0.70, LTS ≥ 7.4°) [11, 14]. Normality was assessed using the Shapiro–Wilk method. Continuous data were recorded as mean and standard deviation if parametric or as median and interquartile range (IQR) if nonparametric. *T*‐tests were used for parametric data, whereas Wilcoxon Rank‐Sum tests were used for nonparametric data. Categorical variables were recorded as frequency and percentage. A *p*‐value of <0.05 was considered statistically significant for all analyses. All statistical analyses were performed using R statistical software (R Foundation for Statistical Computing).

## RESULTS

Of 316 registry records assessed, 26 were excluded, leaving 290 patients for the 2‐year Kaplan–Meier analysis (Figure 2). Twelve were excluded due to no recorded postoperative follow‐up. Among included patients, mean total available follow‐up was 4.4 ± 1.6 years, and all had at least 2 years of follow‐up. Baseline demographic and clinical characteristics are presented in Table 1.

**Figure 2:**
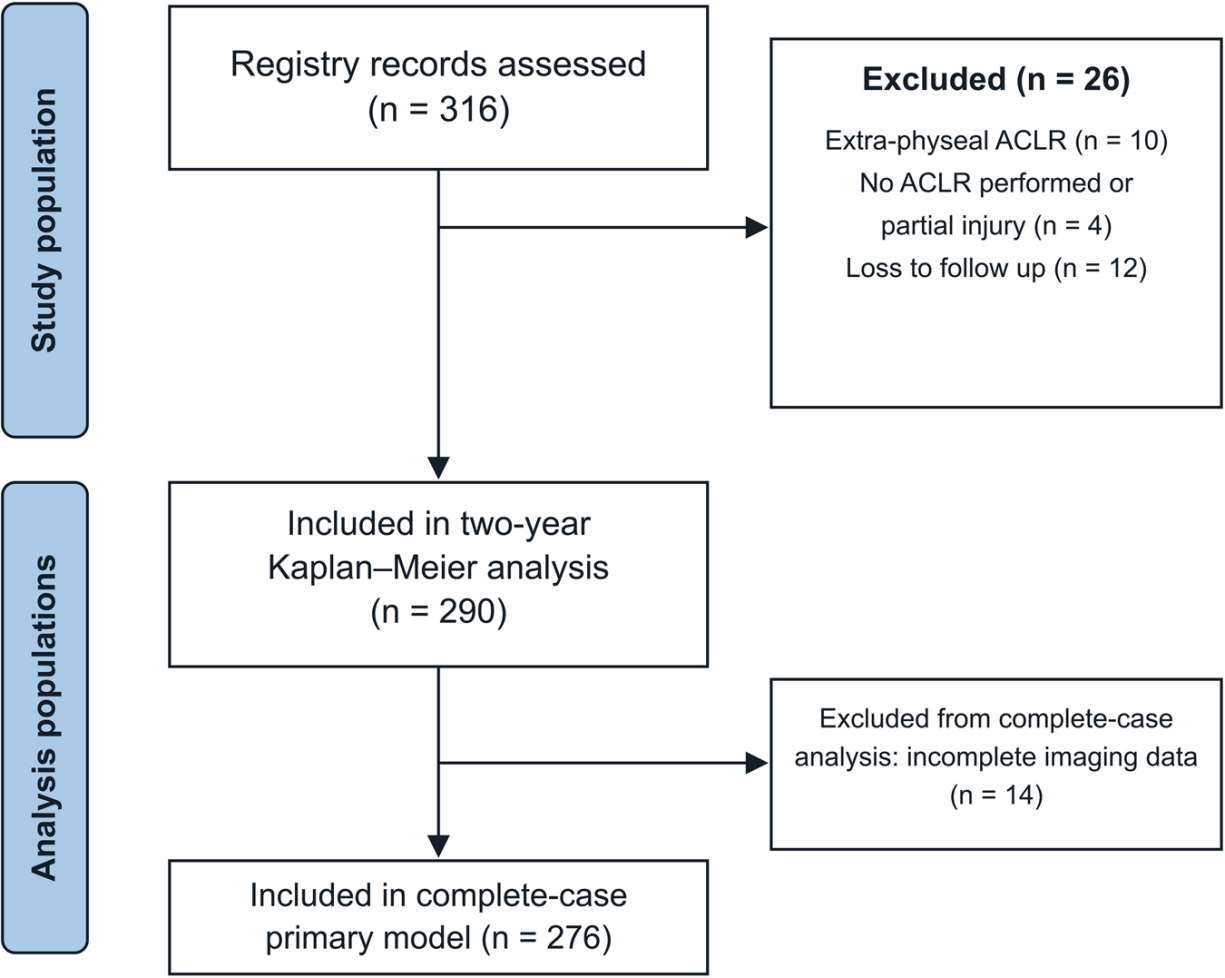
Participant flow diagram. ACLR, anterior cruciate ligament reconstruction.

Within the 2‐year analysis period, 20 graft failure events occurred, with a mean time to failure of 13.4 ± 6.1 months. Of the 20 primary outcome events, 18 were confirmed graft ruptures and two were clinical failures with persistent instability requiring repeat surgery despite an intact graft. Of the 18 graft ruptures, 16 underwent revision ACLR and 2 were managed conservatively. One clinical failure involved recurrent symptomatic instability with a GNRB® side‐to‐side difference >5 mm and was treated with revision ACLR. The second involved persistent symptomatic instability despite an intact graft and was treated with additional LET. An additional five patients demonstrated a GNRB® arthrometer side‐to‐side difference >5 mm, with a median side‐to‐side difference of 5.2 mm (IQR: 5.1–5.7 mm). None of these patients reported subjective instability. Four additional patients demonstrated a grade‐2 pivot shift at the 12‐month assessment; however, all had a GNRB® side‐to‐side difference <5 mm and none reported subjective instability. As these patients did not have symptomatic instability requiring operative treatment, they were not classified as graft failures.

Repeat procedures for other causes within 2 years included eight secondary meniscal procedures. Two patients underwent examination under anaesthesia and arthroscopic adhesiolysis for stiffness, one patient underwent debridement of a cyclops lesion, and one underwent arthroscopic washout for a suspected infection, later confirmed to be noninfective synovitis. Therefore, the overall repeat surgery rate for any indication was 10.34% (*n* = 30).

Fourteen cases were excluded from modelling due to incomplete imaging data, leaving 276 for the final model. None of the excluded cases were from the graft failure group. No statistically significant association between missingness and failure status was detected for any candidate variable (all *p* > 0.05), however, these analyses do not exclude bias from missing data. Overall 2‐year graft survival probability estimated using Kaplan–Meier methods was 93.1% (95% CI: 90.2%–96.1%) (Figure 3). On univariable Cox regression analysis, lower LFCI (HR per 0.01‐unit increase: 0.941, 95% CI: 0.890–0.995, *p* = 0.032), greater LTS (HR: 1.12, 95% CI: 1.00–1.25, *p* = 0.044), higher BMI (HR: 1.12, 95% CI: 1.04–1.22, *p* = 0.003) and medial meniscal compromise (HR: 3.09, 95% CI: 1.12–8.51, *p* = 0.029) were associated with graft failure. No significant associations were observed for age, sex, skeletal age, LET, MAD, MTS or lateral meniscal compromise (Table 2). Based on the twenty graft‐failure events and a prespecified minimum of ten events per variable, the primary model was restricted to two variables. Among the evaluated two‐variable candidate models, the model containing BMI and LFCI had the lowest AIC (199.59) and was selected as the primary multivariable model. In the primary multivariable model, similar findings were observed, where lower LFCI (HR per 0.01‐unit increase: 0.93, 95% CI: 0.88–0.99, *p* = 0.018) and higher BMI (HR: 1.13, 95% CI: 1.05–1.22, *p* = 0.001) remained associated with graft failure. No evidence of violation of the proportional hazards assumption was detected for the primary model using Schoenfeld residuals (global *p* = 0.24). The model had an apparent C‐index of 0.73 in this cohort. This in‐sample estimate should be interpreted cautiously given the limited number of events and data‐informed model selection; validated predictive performance has not been established. The overall likelihood ratio test was statistically significant (*p* < 0.001). Profile‐likelihood CIs were also calculated and were consistent with the Wald CIs.

**Figure 3:**
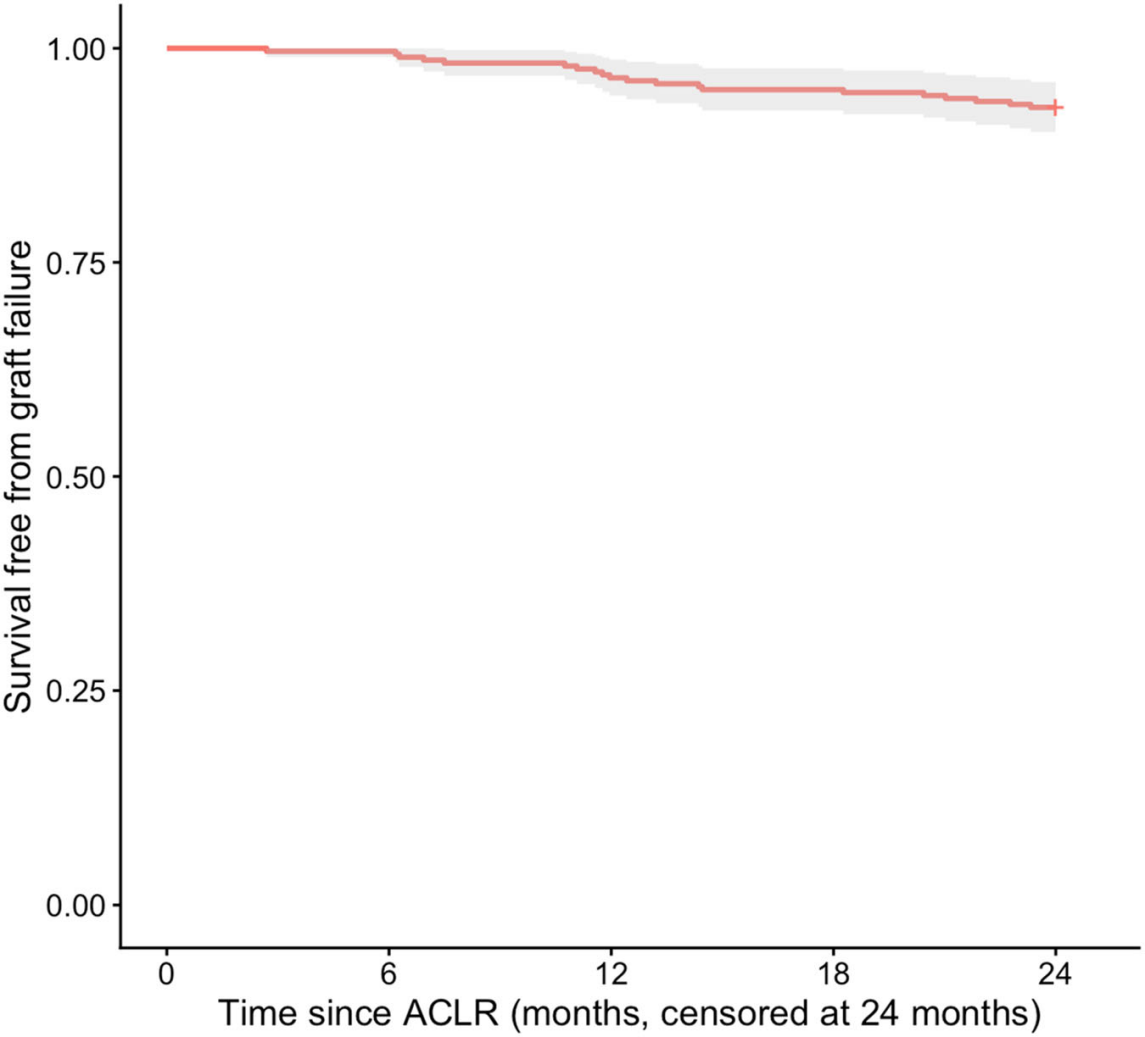
Kaplan–Meier graft survival curve following adolescent anterior cruciate ligament reconstruction. Kaplan‐Meier estimate of survival free from graft failure in 290 patients who underwent primary anterior cruciate ligament reconstruction (ACLR), censored at 2 years. Shaded region denotes the 95% confidence interval. The estimated 2‐year graft survival probability was 93.1%.

**Table 2:** Univariable and multivariable Cox proportional hazards regression for graft failure.

Variable	Univariable HR (95% CI)	*p*‐Value	Multivariable HR (95% CI)	*p*‐Value
BMI (per kg/m^2^ increase)	1.12 (1.04–1.22)	**0.003**	1.13 (1.05–1.22)	**0.001**
LFCI (per 0.01‐unit increase)	0.941 (0.890–0.995)	**0.032**	0.93 (0.88–0.99)	**0.018**
Medial meniscal compromise (yes vs. no)	3.09 (1.12–8.51)	**0.029**	—	—
LTS (per ° increase)	1.12 (1.00–1.25)	**0.044**	—	—
Less than 12 months skeletal growth remaining, estimated preoperatively (yes vs. no)	4.01 (0.93–17.30)	0.062	—	—
Age (per year increase)	1.19 (0.89–1.59)	0.237	—	—
MTS (per ° increase)	1.08 (0.95–1.23)	0.239	—	—
Lateral meniscal compromise (yes vs. no)	1.75 (0.67–4.54)	0.254	—	—
LET (yes vs. no)	1.42 (0.51–3.99)	0.504	—	—
MAD (per mm)	1.01 (0.97–1.06)	0.551	—	—
Sex (female vs. male)	0.77 (0.32–1.86)	0.561	—	—

*Note*: Multivariable Cox regression included BMI and LFCI. The model had an apparent C‐index of 0.73 in this cohort; validated predictive performance has not been established. The overall likelihood ratio test was statistically significant (*p* < 0.001). Dashes indicate variables not included in the multivariable model. Hazard ratios are presented with Wald 95% confidence intervals. Profile‐likelihood confidence intervals were also calculated and yielded consistent conclusions. Bold *p*‐values indicate statistical significance (*p* < 0.05).

Abbreviations: BMI, body mass index; CI, confidence interval; HR, hazard ratio; LET, lateral extra‐articular tenodesis; LFCI, lateral femoral condyle index; LTS, lateral tibial slope; MAD, mechanical axis deviation; MTS, medial tibial slope.

In the rupture‐only sensitivity analysis of 276 patients with 18 confirmed graft ruptures, higher BMI (HR: 1.14, 95% CI: 1.05–1.23; *p* = 0.001) and lower LFCI (HR per 0.01‐unit increase: 0.915, 95% CI: 0.859–0.974; *p* = 0.005) remained associated with graft rupture. Given the potential for skeletal growth to confound the observed associations, an exploratory sensitivity analysis additionally adjusted the primary model for skeletal maturity. The associations of higher BMI (HR: 1.11, 95% CI: 1.02–1.20; *p* = 0.011) and lower LFCI (HR per 0.01‐unit increase: 0.919, 95% CI: 0.865–0.977; *p* = 0.006) with graft failure were maintained following adjustment for skeletal growth. The estimate for skeletal growth was imprecise (HR: 3.27, 95% CI: 0.71–15.12; *p* = 0.129). Substituting raw BMI with age‐ and sex‐standardised BMI z‐score yielded comparable findings. BMI z‐score remained associated with graft failure in the sensitivity model (HR: 1.77 per standard deviation, 95% CI: 1.18–2.64; *p* = 0.006) and the association between LFCI and failure remained unchanged (HR per 0.01‐unit increase: 0.94, 95% CI: 0.88–0.99; *p* = 0.024). These findings demonstrate that the BMI association remains directionally consistent with age‐sex standardisation.

Kaplan–Meier survival curves were generated for morphological and clinical subgroups of interest (Figure 4). An LFCI less than 0.70 has been defined as a cutoff for both index ACL injury and graft failure, and within this cohort, patients with an LFCI below 0.70 demonstrated visibly inferior graft survival compared to those with an LFCI of 0.70 or above [14]. However, this difference did not reach statistical significance (*p* = 0.10), likely reflecting limited power within this dichotomised subgroup. Stratification by a LTS threshold of 7.4° demonstrated significantly reduced graft survival in patients with steeper LTS (*p* = 0.021) [11]. This threshold was derived from MRI‐based measurements, consistent with the imaging modality used in the present cohort. Patients with medial meniscal compromise demonstrated a trend towards worse graft survival compared to those without medial meniscal compromise (*p* = 0.059).

**Figure 4:**
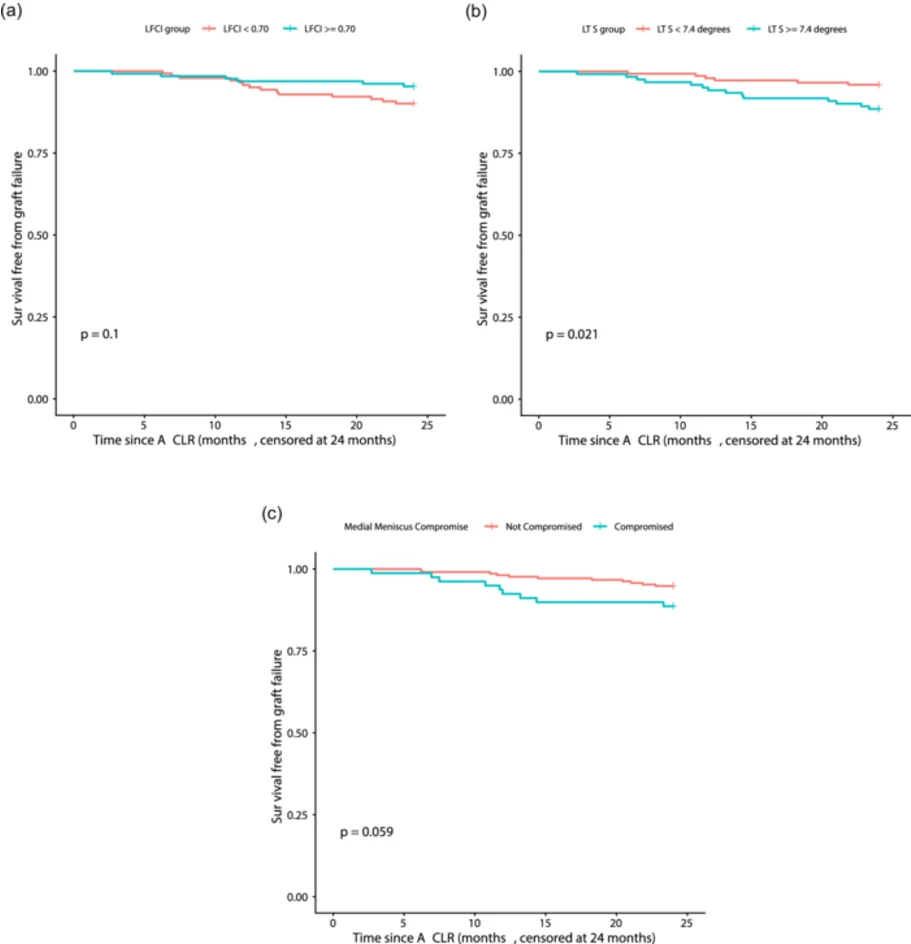
Exploratory Kaplan‐Meier graft survival curves stratified by lateral femoral condyle index (a), lateral tibial slope (b) and medial meniscal compromise (c). (a) Graft survival stratified by lateral femoral condyle index (LFCI) threshold of 0.70 defined by Hodel et al. [14]. Patients with LFCI < 0.70 demonstrated inferior graft survival compared with those with LFCI ≥ 0.70, though this did not reach statistical significance on log‐rank testing (*p* = 0.10). (b) Graft survival stratified by lateral tibial slope (LTS) threshold of 7.4 degrees measured on magnetic resonance imaging, as defined by Grassi et al. [11]. Patients with LTS ≥ 7.4° demonstrated inferior graft survival compared with those with LTS < 7.4° (*p* = 0.021). (c) Graft survival stratified by medial meniscal status. Patients with medial meniscal compromise demonstrated a trend towards worse graft survival compared to those without medial meniscal compromise (*p* = 0.059).

Measurement reliability was excellent, with intra and interrater ICCs (95% CI) of 0.95 (0.92–0.97) and 0.91 (0.83–0.99) for LFCI, 0.99 (0.99–1.00) and 0.99 (0.98–1.00) for MAD, 0.93 (0.90–0.95) and 0.96 (0.82–0.99) for LTS and 0.94 (0.91–0.97) and 0.91 (0.84–0.98) for MTS, respectively [23].

## DISCUSSION

This study explored factors associated with graft failure within 2 years of ACLR in a cohort of 290 adolescent patients, recording 20 failure events and an overall failure rate of 6.90%. On multivariable Cox proportional hazards analysis, higher BMI and lower LFCI were associated with graft failure. These associations were maintained in exploratory sensitivity analyses restricted to confirmed structural graft ruptures and after additional adjustment for skeletal maturity. Medial meniscal compromise and greater LTS were associated with failure on univariable analysis but could not be robustly assessed in the primary multivariable model because of the limited number of events. MAD, MTS, lateral meniscal compromise, sex, skeletal age and the addition of LET were not associated with failure on univariable analysis. Given the limited number of failure events, these findings should be interpreted as exploratory associations rather than definitive prognostic factors.

The observed 2‐year graft failure incidence of 6.9% in this adolescent cohort compares favourably with prior series involving similarly aged cohorts. A systematic review and meta‐analysis of patients 19 years and younger reported pooled autograft failure rates of 10%–15% [4]. A 10‐year single‐centre review of 561 cases of paediatric and adolescent ACLR reported a failure rate of approximately 9.6% with an average time to failure of 13.6 months, though this cohort included a substantial proportion of bone‐patellar tendon‐bone grafts, which demonstrated superior survival compared to hamstring autografts in that series [13]. In this exclusively hamstring‐autograft cohort, the observed graft survival suggests that acceptable outcomes can be achieved in adolescents. However, the absence of a comparator graft group precludes conclusions regarding the independent effect of graft type on failure risk. Higher BMI and BMI *z*‐scores were associated with failure in this study. Each one‐unit increase in BMI conferred approximately a 13% increase in hazard. Raw BMI was selected over BMI *z*‐score because graft loading is more directly related to absolute body mass relative to height than population‐standardised adiposity. Notably, this association persisted after adjustment for LFCI, which highlights the importance of exploring the interaction between BMI and bony morphology in future studies. Although derived from a mixed adolescent and adult cohort, registry data from the Swedish and Norwegian Knee Ligament Registries demonstrated that a BMI ≥ 25 kg/m^2^ was associated with an increased risk of early ACL revision, with similar associations observed in the adolescent subgroup [25]. Preoperative skeletal maturity was not significantly associated with graft failure in the present cohort, despite reaching significance in the unadjusted comparison in Table 1 (*p* = 0.044). Importantly, skeletal maturity was assessed from preoperative MRI and used to estimate whether patients had greater than 12 months of remaining skeletal growth; actual growth following ACLR was not prospectively measured. This distinction may be clinically relevant, as Bartek et al. reported significantly greater postoperative height increase among skeletally immature patients who subsequently underwent revision ACLR compared with those with an intact reconstruction (12 ± 8.7 cm vs. 5 ± 4.3 cm), with increasing height also associated with greater anterior tibial translation [1]. Therefore, the absence of an association with preoperative estimated remaining growth in the present study does not exclude a potential relationship between actual postoperative growth and graft failure. Nevertheless, additional adjustment for preoperative skeletal maturity in an exploratory sensitivity analysis did not materially alter the associations between BMI, LFCI and graft failure.

Among knee morphological parameters, lower LFCI was associated with graft failure in both univariable and multivariable models, such that each 0.01‐unit decrease in LFCI conferred a 6%–7% increase in failure hazard. A lower LFCI reflects a relatively smaller posterior lateral femoral condyle and may reduce osseous constraint during anterior translation and internal rotation [14]. Greater LTS was associated with graft failure in univariable analysis but was not included in the selected primary multivariable model. Overall, each one‐degree increase in LTS increased the hazard of failure by approximately 12%. This finding is consistent with previous literature demonstrating increased posterior tibial slope as a risk factor for both primary ACL injury and graft failure [5, 7, 9, 11]. Biomechanically, increased tibial slope is thought to increase anterior tibial translation and ACL graft loading during dynamic activities, thereby increasing susceptibility to reinjury. MTS did not demonstrate significant findings within this study. This is consistent with studies suggesting that the lateral compartment may have a greater influence on pivoting mechanics and rotational instability than the medial compartment [11]. Furthermore, despite prior studies demonstrating associations between lower LFCI, higher LTS and valgus alignment in paediatric patients with ACL rupture [23], coronal plane alignment using MAD was not associated with graft failure in this study. Therefore, MAD may not completely reflect the complex rotational biomechanics of the knee joint. Nevertheless, the interaction between morphological measures and graft outcomes requires validation in large studies.

For analysis, meniscal compromise was defined as a tear managed with debridement or no intervention. This exploratory grouping was intended to capture possible tissue loss or persistent meniscal pathology, although debrided and untreated tears are clinically distinct. Table 1 therefore reports intact, repaired, debrided and untreated menisci separately. Nevertheless, medial meniscal compromise was associated with a higher graft failure hazard on univariable analysis (HR: 3.09), but the small number of events prevented a reliable assessment of its independent association. No significant association was observed for binary lateral meniscal compromise in Cox analysis. The finding should be interpreted in light of the composite definition and warrants further study using separate meniscal categories. Previous studies in adults have identified both medial and lateral meniscal deficiency, defined as any meniscal resection, as independent predictors of graft failure [19]. The menisci are important secondary stabilisers of the knee joint and meniscal tears increase forces on both native and reconstructed ACL under rotational loading [27]. The addition of LET had a positive but nonsignificant hazard ratio in the current cohort (HR: 1.42; *p* = 0.504). Given its preferential use in higher‐risk patients within this cohort, this estimate may reflect confounding by indication rather than a harmful effect of LET, a methodological consideration that the STABILITY trial was specifically designed to address through randomisation [8]. Accordingly, the current data neither support nor refute a beneficial effect of LET on graft survival in this population, and the limited number of LET cases further constrains statistical power to detect a meaningful difference. The STABILITY randomised trial demonstrated a reduction in graft rupture rates with the addition of LET to hamstring autograft ACL reconstruction [8]. The observed graft failure rate should be interpreted in the context of selective LET use alongside hamstring autograft ACL reconstruction in patients considered at higher risk of failure. Given that the longer‐term effects of contemporary LET procedures remain incompletely characterised in this age group, its use should be individualised according to patient‐specific risk factors until further comparative evidence becomes available.

These findings have several potential implications for the management of adolescent ACL injuries. Morphological factors including both LFCI and LTS could be considered during preoperative assessment. The observed associations of higher BMI and lower LFCI with graft failure warrant further investigation in larger cohorts. These exploratory findings do not establish whether modifying surveillance, rehabilitation or return‐to‐sport protocols according to these characteristics improves outcomes. The exploratory association between medial meniscal compromise and failure requires further study and emphasises the importance of thorough intraoperative meniscal assessment. Future studies should prospectively capture patient‐reported outcome measures and return‐to‐sport data alongside structural failure endpoints. This is in keeping with consensus recommendations for multidomain assessment of ACL outcomes, given that graft integrity alone may not fully reflect the functional recovery and sporting participation that are paramount to this patient population.

There are several limitations of this exploratory study. First, while the 2‐year follow‐up window captures the highest‐risk period for graft failure, inferences about longer‐term graft integrity are limited. Second, morphology was assessed at a single preoperative timepoint across patients at different stages of skeletal development; subsequent growth‐related change may therefore limit measurement precision and generalisability. Third, with only 20 failure events, the study was underpowered to simultaneously adjust for correlated morphological and clinical associations. The prespecified events‐per‐variable threshold restricted the primary multivariable model to two covariates, meaning LTS and medial meniscal compromise could not be assessed independently of BMI and LFCI. Candidate prioritisation and final model selection were informed by the observed data, introducing additional uncertainty. The reported confidence intervals and *p*‐values do not account for this model‐selection process and should therefore be interpreted as exploratory. This same limitation likely explains why the dichotomised Kaplan–Meier comparisons (Figure 4a,c) showed visibly separated but nonsignificant survival curves for LFCI and medial meniscal status. Fourth, although all procedures used hamstring autografts and were performed by fellowship‐trained paediatric orthopaedic surgeons at a single centre, operative technique, graft preparation, fixation and LET use varied according to clinical indications and surgeon preference. These choices may have been related to skeletal maturity, knee morphology and baseline instability, introducing confounding by indication. The limited number of failure events constrained adjustment for these surgical factors and surgeon‐level effects. Consequently, the observed associations cannot be assumed to be independent of surgical management. Restricting the endpoint to confirmed graft rupture addressed outcome‐definition heterogeneity but did not resolve potential confounding from surgical variation. Fifth, complete‐case analysis excluded 14 patients (4.8%) and potential bias from complete‐case exclusion cannot be ruled out. Sixth, return‐to‐sport timing and postoperative sporting exposure were not consistently available and could not be incorporated into the analysis. Consequently, differences in exposure to reinjury risk between patients could not be accounted for and may represent a source of residual confounding. Finally, as the cohort comprised exclusively hamstring autografts performed at a single paediatric centre, findings may not be generalisable to other cohorts incorporating a variety of graft types.

## CONCLUSION

Higher BMI and lower LFCI were associated with 2‐year graft failure following adolescent ACL reconstruction, with these associations maintained in exploratory sensitivity analyses. Given the limited number of failure events, these findings should be considered exploratory and require validation in larger cohorts before application to individual risk prediction.

## AUTHOR CONTRIBUTIONS

**Alexander H. Seeto**: Conceptualisation; data curation; formal analysis; investigation; methodology; project administration; validation; visualisation; writing—original draft; writing—review and editing. **Kylie Bradford**: Data curation; investigation; writing—review and editing. **David Bade**: Data curation; investigation; resources; writing—review and editing. **Liam Johnson**: Data curation; investigation; resources; writing—review and editing. **Sheanna Maine**: Data curation; investigation; resources; writing—review and editing. **David J. Saxby**: Supervision; methodology; writing—original draft; writing —review and editing. **Christopher P. Carty**: Project administration; supervision; methodology; resources; validation; writing—original draft; writing—review and editing. **Ivan P. Astori**: Conceptualisation; principal investigator; project administration; resources; supervision, methodology; writing—original draft; writing—review and editing.

## FUNDING INFORMATION

The authors have no funding to report.

## CONFLICT OF INTEREST STATEMENT

Alexander H. Seeto, David J. Saxby, Kylie Bradford and Liam Johnson have nothing to disclose. Christopher P. Carty and David Bade received grant funding to their institutions from Children's Hospital Foundation (Brisbane) and Smith and Nephew (not related to this current project). Sheanna Maine has received honoraria from Smith and Nephew. Ivan P. Astori has received consulting fees and honoraria from Smith and Nephew and Orthofix.

## ETHICS STATEMENT

Children's Health Queensland Hospital and Health Service Human Research Ethics Committee, HREC/18/QRCH/182. Guardians of all participants provided written informed consent.

## Supporting information

Supplementary material — STROBE checklist.

## Data Availability

The data that support the findings of this study are available on reasonable request from the corresponding author. The data are not publicly available due to privacy or ethical restrictions.
