# Supplementary material for: Higher body mass index and lower lateral femoral condyle index are associated with graft failure following adolescent anterior cruciate ligament reconstruction: A registry‐based 2‐year survival analysis
Source: J Exp Orthop. 2026 Nov 11;13(4):e70978. doi: 10.1002/jeo2.70978 (PMC13754412; doi:10.1002/jeo2.70978)
Supplement: Supplementary file 1 [file JEO2-13-e70978-s001.docx]

**Supplementary material — STROBE checklist**

STROBE Statement—checklist of items that should be included in reports of observational studies

|  | Item No. | Recommendation | Page  No. | Relevant text from manuscript |
| --- | --- | --- | --- | --- |
| **Title and abstract** | 1 | (*a*) Indicate the study’s design with a commonly used term in the title or the abstract | p. 1, lines 1–2 and 10–17 | Title identifies a registry-based survival analysis; abstract Methods describes prospective registry recruitment and time-to-event analysis. |
|  |  | (*b*) Provide in the abstract an informative and balanced summary of what was done and what was found | pp. 1–2, lines 5–31 | Structured abstract reports purpose, cohort and methods, event count, survival estimate, associations, and conclusions. |
| Introduction | | | |  |
| Background/rationale | 2 | Explain the scientific background and rationale for the investigation being reported | p. 3, lines 35–49 | Clinical burden, incomplete evidence on graft-failure risk factors, and rationale for assessing knee morphology. |
| Objectives | 3 | State specific objectives, including any prespecified hypotheses | p. 3, lines 50–57 | Primary aim: describe two-year graft-failure incidence; secondary aim: explore morphological and meniscal associations. An explicit hypothesis concerning patient-specific, morphological and surgical factors is stated. |
| Methods | | | |  |
| Study design | 4 | Present key elements of study design early in the paper | p. 4, lines 60–70 | Prospectively recruited registry cohort of adolescents undergoing primary ACL reconstruction, with two-year graft failure assessed. |
| Setting | 5 | Describe the setting, locations, and relevant dates, including periods of recruitment, exposure, follow-up, and data collection | p. 4, lines 60–70; p. 9, lines 170–181 | Single paediatric ACL registry; recruitment September 2018–January 2024; multimodal follow-up; censoring at two years. |
| Participants | 6 | (*a*) *Cohort study*—Give the eligibility criteria, and the sources and methods of selection of participants. Describe methods of follow-up  *Case-control study*—Give the eligibility criteria, and the sources and methods of case ascertainment and control selection. Give the rationale for the choice of cases and controls  *Cross-sectional study*—Give the eligibility criteria, and the sources and methods of selection of participants | p. 4, lines 60–70; p. 9, lines 170–181 | Eligibility and exclusions, outpatient recruitment, registry source, and electronic-record, imaging, and telephone follow-up are described. |
|  |  | (*b*) *Cohort study*—For matched studies, give matching criteria and number of exposed and unexposed  *Case-control study*—For matched studies, give matching criteria and the number of controls per case | Not applicable. | Not applicable. |
| Variables | 7 | Clearly define all outcomes, exposures, predictors, potential confounders, and effect modifiers. Give diagnostic criteria, if applicable | pp. 4–6, lines 72–126; pp. 8–9, lines 140–169 | Demographic, skeletal, meniscal, surgical, morphological, and graft-failure variables and criteria are defined. |
| Data sources/ measurement | 8* | For each variable of interest, give sources of data and details of methods of assessment (measurement). Describe comparability of assessment methods if there is more than one group | pp. 4–6, lines 72–126; pp. 8–9, lines 154–181 | Registry/record sources, MRI and radiographic measurement protocols, intraoperative meniscal assessment, and failure ascertainment are specified. |
| Bias | 9 | Describe any efforts to address potential sources of bias | p. 5, lines 93–102; pp. 9–11, lines 171–214; pp. 27–28, lines 453–482 | Multimodal event capture, missing-data assessment, parsimonious modelling, sensitivity analyses, and residual-bias limitations are reported. |
| Study size | 10 | Explain how the study size was arrived at | p. 10, lines 196–203 | Sample size was determined by the eligible registry cohort. The ten-events-per-parameter criterion limited primary model complexity to two predictor parameters; it was not a formal power calculation. |

| Quantitative variables | 11 | | Explain how quantitative variables were handled in the analyses. If applicable, describe which groupings were chosen and why | pp. 4–5, lines 78–91; pp. 10–11, lines 187–189 and 214–226 | | Continuous predictors were modelled linearly; skeletal-growth and exploratory morphology thresholds and their sources are specified. | |  |
| --- | --- | --- | --- | --- | --- | --- | --- | --- |
| Statistical methods | 12 | | (*a*) Describe all statistical methods, including those used to control for confounding | pp. 9–11, lines 183–229; Table 1 footnote, p. 17 | | Kaplan–Meier estimation, Cox regression, proportional-hazards checks, complete-case analysis, model selection, confidence intervals, and software are described. | |  |
|  |  |  | (*b*) Describe any methods used to examine subgroups and interactions | p. 11, lines 221–223 | | Exploratory Kaplan–Meier analyses used literature-based LFCI and LTS thresholds and binary medial meniscal compromise. No interaction analyses are reported. | |  |
|  |  |  | (*c*) Explain how missing data were addressed | p. 10, lines 191–194 | | Complete-case analysis was used; associations between missingness and failure were assessed by logistic regression adjusted for age and sex. | |  |
|  |  |  | (*d*) *Cohort study*—If applicable, explain how loss to follow-up was addressed  *Case-control study*—If applicable, explain how matching of cases and controls was addressed  *Cross-sectional study*—If applicable, describe analytical methods taking account of sampling strategy | p. 9, lines 170–181; pp. 11–12, lines 231–235; Figure 2 | | Follow-up was sought through registry records, linked medical records, imaging and telephone contact. Twelve patients without recorded postoperative follow-up were excluded. All included patients had at least two years of follow-up; event-free patients were censored at two years. | |  |
|  |  |  | (*e*) Describe any sensitivity analyses | pp. 10–11, lines 205–220 | | Exploratory sensitivity analyses restricted events to confirmed graft rupture, additionally adjusted the BMI–LFCI model for skeletal maturity, and substituted age- and sex-standardised BMI z-scores for raw BMI. Clinical failures were censored at their date of clinical failure in the rupture-only analysis. | |  |
| Results | | | | | | | |  |
| Participants | 13* | | (a) Report numbers of individuals at each stage of study—eg numbers potentially eligible, examined for eligibility, confirmed eligible, included in the study, completing follow-up, and analysed | pp. 11–12, lines 231–235 and 254–257; Figure 2, p. 16 | | Of 316 registry records assessed, 26 were excluded, leaving 290 participants in the survival analysis. Fourteen participants with incomplete imaging data were excluded from Cox modelling, leaving 276 complete cases. | |  |
|  |  |  | (b) Give reasons for non-participation at each stage | pp. 11–12, lines 231–235 and 254–257; Figure 2, p. 16 | | Exclusions comprised 10 extra-physeal reconstructions, four cases without ACL reconstruction or with partial tears, and 12 without recorded postoperative follow-up. A further 14 participants had incomplete imaging data for modelling. | |  |
|  |  |  | (c) Consider use of a flow diagram | Figure 2, p. 16 | | Figure 2 presents participant screening, exclusions and the cohorts included in survival analysis and complete-case modelling. | |  |
| Descriptive data | 14* | | (a) Give characteristics of study participants (eg demographic, clinical, social) and information on exposures and potential confounders | Table 1, pp. 17–18 | | Baseline demographic, surgical, meniscal, and morphological characteristics are reported overall and by graft-failure status. | |  |
|  |  |  | (b) Indicate number of participants with missing data for each variable of interest | p. 12, lines 254–257 | | Fourteen participants had incomplete modelling data; none were in the graft-failure group; missingness was not associated with failure. | |  |
|  |  |  | (c) *Cohort study*—Summarise follow-up time (eg, average and total amount) | pp. 11–12, lines 233–235 | | Mean total available follow-up was 4.4 ± 1.6 years; all participants had at least two years of follow-up. | |  |
| Outcome data | 15* | | *Cohort study*—Report numbers of outcome events or summary measures over time | pp. 12–13, lines 236–259; Figure 3, p. 19 | | Twenty graft failures occurred within two years; event types, revision procedures, repeat surgery, and two-year survival are reported. | |  |
|  |  |  | *Case-control study—*Report numbers in each exposure category, or summary measures of exposure | Not applicable | | Not applicable | |  |
|  |  |  | *Cross-sectional study—*Report numbers of outcome events or summary measures | Not applicable | | Not applicable | |  |
| Main results | 16 | | (*a*) Give unadjusted estimates and, if applicable, confounder-adjusted estimates and their precision (eg, 95% confidence interval). Make clear which confounders were adjusted for and why they were included | pp. 13–14, lines 259–289; Table 2, p. 20 | | Univariable and adjusted hazard ratios with 95% CIs are reported; BMI and LFCI constitute the primary adjusted model. | |  |
|  |  |  | (*b*) Report category boundaries when continuous variables were categorized | p. 11, lines 221–223; p. 14, lines 290–299; Figure 4, pp. 21–23 | | Reported subgroup boundaries were LFCI 0.70 and LTS 7.4°, with medial meniscal compromise defined categorically. | |  |
|  |  |  | (*c*) If relevant, consider translating estimates of relative risk into absolute risk for a meaningful time period | p. 13, lines 258–259; Figure 3, p. 19 | | Two-year graft survival was 93.1% (95% CI 90.2–96.1%). | |  |
| Other analyses | 17 | Report other analyses done—eg analyses of subgroups and interactions, and sensitivity analyses | | | pp. 13–14, lines 277–299; Figure 4, pp. 21–23 | | Rupture-only, skeletal-maturity-adjusted and BMI z-score sensitivity analyses, together with exploratory subgroup Kaplan–Meier analyses, are reported.. | |
| Discussion | | | | | | | |  |
| Key results | 18 | Summarise key results with reference to study objectives | | | p. 24, lines 352–362; p. 28, lines 485–488 | | The Discussion and Conclusion summarise two-year graft failure and exploratory associations of BMI and LFCI, including their persistence in sensitivity analyses.. | |
| Limitations | 19 | Discuss limitations of the study, taking into account sources of potential bias or imprecision. Discuss both direction and magnitude of any potential bias | | | pp. 27–28, lines 453–482 | | Limitations include follow-up duration, growth-related changes, limited events, data-informed model selection, surgical confounding, complete-case exclusion, unavailable sporting exposure and limited generalisability. | |
| Interpretation | 20 | Give a cautious overall interpretation of results considering objectives, limitations, multiplicity of analyses, results from similar studies, and other relevant evidence | | | pp. 24–28, lines 352–488 | | Findings are interpreted cautiously against prior evidence, mechanisms, limited power, confounding by indication, and clinical implications. | |
| Generalisability | 21 | Discuss the generalisability (external validity) of the study results | | | p. 27, lines 454–457; p. 28, lines 480–482 | | External validity is discussed for skeletal development, the single paediatric centre, and the exclusively hamstring-autograft cohort. | |
| Other information |  | | | | | | |  |
| Funding | 22 | Give the source of funding and the role of the funders for the present study and, if applicable, for the original study on which the present article is based | | | Separate title page: Funding declaration | | The separate title page states: “No funding was received for this work.”. | |

*Give information separately for cases and controls in case-control studies and, if applicable, for exposed and unexposed groups in cohort and cross-sectional studies.

**Note:** An Explanation and Elaboration article discusses each checklist item and gives methodological background and published examples of transparent reporting. The STROBE checklist is best used in conjunction with this article (freely available on the Web sites of PLoS Medicine at http://www.plosmedicine.org/, Annals of Internal Medicine at http://www.annals.org/, and Epidemiology at http://www.epidem.com/). Information on the STROBE Initiative is available at www.strobe-statement.org.
